# The mediating role of psychological resilience in the relationship between frailty and self-efficacy among dialysis patients

**DOI:** 10.3389/fpsyt.2025.1542031

**Published:** 2026-01-12

**Authors:** Su Fang Jiang, Kang Ning Wang, Shan Zhang, Yu Rong Zhang, Man Zhen Dong, Qi Qi Li, Nan Hui Zhang, Juan Juan Lin, Long Hua Rao

**Affiliations:** 1 Department of Nephrology, Hemodialysis Unit, Xiangyang No.1 People’s Hospital, Hubei University of Medicine, Xiangyang, China; 2 Department of Neurosurgery, Xiangyang Central Hospital, Xiangyang, China; 3 Department of Operating Theatre, Xiangyang Central Hospital, Xiangyang, China; 4 Department of Nursing, Xiangyang No.1 People’s Hospital, Hubei University of Medicine, Xiangyang, China

**Keywords:** dialysis, frailty, mediating effect, psychological resilience, self-efficacy

## Abstract

**Background:**

Frailty poses a substantial challenge for patients with hemodialysis (HD), influenced by a multitude of personal and social factors. Building on the theory of Hobfoll’s Conservation of Resources (COR) theory, this study sought to evaluate the correlation between frailty and self-efficacy, as well as psychological resilience among patients undergoing HD treatment.

**Methods:**

A cross-sectional study was conducted on 397 HD patients at various hospitals in Xiangyang City, Hubei Province, China. Data was gathered between February and May, 2024 using the Frail Scale, the Connor-Davidson Resilience Scale (CD-RISC), and the self-efficacy scale. The collected data were analyzed using SPSS software, applying both descriptive and inferential statistical methods, as well as conducting mediation effect analysis and structural equation modeling with bootstrapping by Amos 26.0.

**Results:**

The study revealed that the prevalence of pre-frailty and frailty was 26.2% and 38.3%, respectively. The self-efficacy score of the patients was 6.5 (5.0, 8.2) on a scale from 0 to 10. Additionally, the psychological resilience score was 23.0 (20.0, 30.0) on a scale from 0 to 40. The results indicated correlations among psychological resilience, frailty, and self-efficacy in HD patients. Frailty had a negative correlation with self-efficacy (r = -0.166, *p* < 0.01) and psychological resilience (r = -0.222, *p* < 0.01). Conversely, self-efficacy and psychological resilience were positively related (r = 0.287, *p* < 0.01). Psychological resilience, acting as a mediating factor, exhibited an indirect effect size of 35%, while the direct effect size of patient frailty was 65%.

**Conclusion:**

The findings of this study underscore a negative association between frailty and self-efficacy. Furthermore, psychological resilience plays an acceptable mediating role in the relationship between frailty and self-efficacy.

## Background

End-stage renal disease (ESRD) is a typical chronic, irreversible, life-threatening, costly, and disabling illness with a high mortality rate (1, 2). Studies have reported that the unadjusted 5-year survival rate for ESRD patients on kidney replacement therapy (KRT) was 41% in the US, 48% in Europe, and 60% in Japan (3). There are three primary forms of KRT: long-term hemodialysis (HD), continuous ambulatory peritoneal dialysis, and kidney transplantation, all of which generally extend the lives of ESRD patients. It is estimated that the number of ESRD patients requiring KRT will increase from 2.6 million in 2010 to 5.4 million in 2030 worldwide (4). Over the next five years, the number of dialysis patients in China is projected to reach nearly 900,000 cases (5), and it has emerged as one of the most critical public health issues globally (6).

Among ESRD patients on HD, frailty is prevalent and a robust independent predictor of mortality and hospitalizations (7). HD patients experience a decline in renal function and metabolic disorders. The accumulation of toxic substances can trigger inflammation, oxidative stress, cellular aging, sarcopenia/muscle wasting, cognitive impairment, osteoporosis, vascular calcification, cardiopulmonary deconditioning, and more. These conditions may elevate the risk of frailty (8). Furthermore, HD-related symptoms, such as imbalance syndrome, hypotension, nutritional loss, and fatigue, may exacerbate frailty.

Patients with ESRD experience a significant symptom burden, which often leads to reduced quality of life (9, 10). Those undergoing HD must visit hospitals or dialysis centers 2–3 times weekly for 3–4 hour treatments, severely limiting their daily activities and impacting both their social and professional lives (11). Additionally, they must also adhere to stringent medical advice, including dietary restrictions, maintaining fluid balance, consistent medication use, and regular treatment visits (12). These requirements can further contribute to life dissatisfaction and a reduction in their quality of life. To improve HD patients’ quality of life, ensuring their treatment adherence and guiding a healthy lifestyle is vital. Patient care quality is influenced by personal factors such as self-efficacy and psychological resilience, as well as social factors including family resilience and the level of social support.

Self-efficacy is one of the most critical personal attributes among HD patients, reflecting their belief in achieving goals. Patients with elevated self-efficacy generally experience superior overall health outcomes (13), including improved cognitive and emotional function, lower mortality and hospitalization rates, better daily activities and treatment adherence, and enhanced quality of life (14–17). Furthermore, research conducted in Turkey indicated that kidney transplant patients exhibit a moderate level of self-efficacy perception (18). Past studies also indicated that HD patients in Iran exhibit high levels of self-efficacy (14), while dialysis patients in Korea demonstrated a moderate level of self-efficacy (19). Consequently, identifying factors associated with the self-efficacy of these patients appears to be essential.

Psychological resilience has been identified as a protective factor against frailty in HD patients (20). Individual resilience (RES) can alleviate the strain imposed by the adverse effects of chronic diseases (21). RES is characterized as the capacity of a person to effectively endure and adapt to stressors, challenges, or environmental changes (22, 23). Various biological, psychological, social, and cultural factors can impact an individual’s RES (24). Enhanced RES is crucial in alleviating stress experienced during illness, physical disability and stress-related harm (22). Health-promoting behaviors have been utilized to bolster RES in CKD patients, and improved RES can assist them in managing stress and depression, empowering them to lead a more positive life (1, 21). Consequently, boosting RES enables these patients to better handle stress, anxiety, psychological challenges, improving their quality of life.

Based on the literature review, there is scant research on psychological resilience and self-efficacy among HD patients. Furthermore, no study has explored the interaction between self-efficacy, frailty, and psychological resilience in dialysis patients. This study aims to evaluate the correlation between self-efficacy, frailty, and psychological resilience support in HD patients.

## Conceptual framework

Hobfoll’s (1989) Conservation of Resources Theory (COR) focuses on how individuals acquire, keep, and conserve resources (25). Resources in COR are things or means that individuals find valuable or that help them get valuable things, and they fall into four categories: material (e.g., cars, real estate, job tools), conditional (e.g., marriage, position, qualifications), individual trait (e.g., high IQ, self-efficacy, optimism), and energy (e.g., time, money, knowledge). Resource loss can trigger a chain reaction, causing further loss and increasing stress, while active resource acquisition creates a positive cycle and boosts stress coping. COR theory is widely used to explain burnout (26, 27), work environment (28), posttraumatic adaptation (29), and resilience (30).

**Figure 1:**
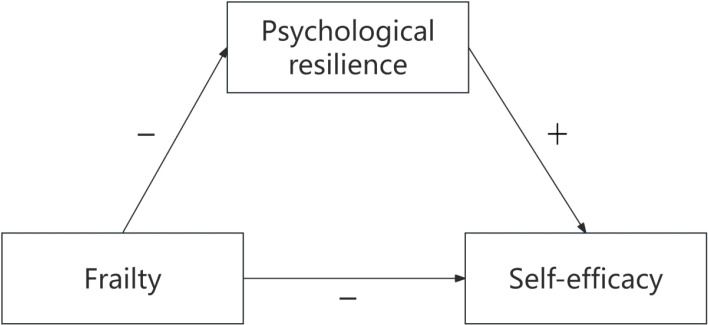
Hypothesized conceptual model.

Frailty, like physical decline, is a resource loss that reduces self-efficacy (confidence in coping). Psychological resilience, a key personal trait resource, helps individuals reduce resource loss impact or buffer frailty’s negative effect on self-efficacy by seeking new resources (e.g., positive coping, social support). Resilient people can maintain or restore confidence despite debility, weakening the negative link between frailty and self-efficacy. The theoretical hypothesis model is shown in **Figure 1**. Our research hypotheses are as follows:

Hypothesis 1: Frailty is negatively and directly related to psychological resilience.
Hypothesis 2: Frailty is negatively and directly related to self-efficacy.
Hypothesis 3: Psychological resilience is positively and directly related to self-efficacy.
Hypothesis 4: There is a mediating effect of psychological resilience between frailty and self-efficacy.

## Conceptual model

Mediation modeling offers a statistical framework for researchers to examine how independent variables indirectly influence dependent variables through mediating variables. In this study, the following hypotheses were made: the independent variable was frailty, the dependent variable was self-efficacy, and psychological resilience functioned as the mediating variable. The mediation model employed in this research is grounded in Hobfoll’s COR Theory. This theory underscores the significance of resource acquisition and depletion in relation to stress levels and resilience. Overall, frailty first erodes psychological resilience by depleting resources, and then, through this weakened resilience, indirectly leads to reduced self-efficacy. Put simply, debilitation undermines psychological resilience, which subsequently lowers self-efficacy.

## Study design and participants

This study utilized a descriptive cross-sectional approach conducted across various dialysis units. The recruitment of participants took place between February 1, 2024, and May 1, 2024, involving 397 maintenance HD patients from various hospitals in Xiangyang City, Hubei Province, China. These hospitals included one Grade III Level-A, one Grade III Level-B, one Grade III comprehensive, and one Grade II Level-A facility.

## Sampling strategy and criteria

Convenience sampling was employed in this cross-sectional study. The inclusion criteria were established to guarantee the participants’ qualifications, encompassing: (1) An age of 18 years or older; (2) possession of clear consciousness, normal cognitive function, and absence of communication barriers; (3) undergoing outpatient HD therapy for a minimum of three months; and (4) voluntary consent to participate in the survey. On the other hand, the exclusion criteria omitted individuals who were unconscious, had dementia, experienced mental illness, or encountered difficulties in communication.

## Sample size estimation

Given the utilization of Structural Equation Modeling (SEM) analysis and the number of questionnaire items and variables under investigation, a minimum of 5 participants per item was considered. Taking into account participant attrition and sample size requirements, a minimum sample size of 215 individuals was determined.

## Assessment of frailty

To assess frailty among the participants, the Simple Frail Scale (31) was employed. This scale encompasses five self-reported questions, each of which is scored as 1 for a “yes” response and 0 for a “no” response, yielding a total score ranging from 0 to 5. The interpretation of scores is as follows: a score of 0 signifies robustness, scores of 1 to 2 indicate pre-frailty, and scores of 3 or more denote frailty. This scale has been validated as an efficient and user-friendly method for screening frailty in HD patients (32). In this study, the Cronbach’s alpha coefficient was 0.824, demonstrating adequate reliability and validity.

## Measurement of psychological resilience

The Connor-Davidson Resilience Scale (CD-RISC) (33), formulated by Connor and Davidson in 2003, serves as a reliable instrument for assessing psychological resilience. The simplified Chinese version of this questionnaire comprises 10 items, with respondents rating each item on a 4-point scale ranging from 0 to 4. The cumulative total score represents the level of psychological resilience, where a higher score indicates greater resilience. The scale has demonstrated high levels of both validity and reliability when applied to a Chinese population, including HD (34, 35). In this study, the Cronbach’s alpha coefficient was 0.964, indicating strong reliability and validity.

## Measurement of self-efficacy

A short version, the self-efficacy scale (36), was developed by Lorig et al. from the Chronic Disease Education Research Center at Stanford University. This scale has a Cronbach’s alpha coefficient of 0.91 and consists of 6 items, divided into two dimensions: symptom management (4 items) and disease commonality management (2 items). Each item is scored on a 1–10 point scale, with 1 point representing “completely no confidence” and 10 points representing “absolutely confident”. The average score of the 6 items constitutes the self-efficacy score. A self-efficacy score of ≥7 indicates a high level, a score of ≥5 and <7 indicates a moderate level, and a score <5 indicates a low level of self-efficacy. The scale has exhibited validity and reliability in the context of a Chinese HD population (37). In this study, the Cronbach’s alpha coefficient was 0.966, indicating strong reliability and validity.

## Data collection on covariates

A comprehensive set of covariate data was collected to provide a thorough understanding of the study participants. Demographic variables recorded included age (categorized as ≥60 years and <60 years), sex (male/female), body mass index (BMI, kg/m (2)), marital status (married, single, divorced, widowed), education level (elementary school and below/above elementary school), residential situation (living with family/others), employment status (yes/no), and monthly household income (≤3000 yuan/>3000 yuan). Lifestyle factors considered were smoking status (smokers, quit smokers, and never smokers), drinking status (drinkers, quit drinkers, and never drinkers), and the regularity of exercise (yes/no).

Additionally, factors related to dialysis treatment were included in the data collection. These comprised dialysis duration (categorized as <5 years and ≥5 years), dialysis frequency (twice a week, three times a week, and five times in two weeks), occurrence of falls (yes/no), type of vascular access (arteriovenous fistula, artificial blood vessels, and central venous catheter), presence of hypertension (yes/no), diabetes (yes/no), heart disease (yes/no), cerebral disease (yes/no), and existence of other comorbidities (yes/no). **Table 1** outlines the operationalization of these variables.

**Table 1:** Assignment of explanatory variables.

Variables	Description
Sex	Male=1,Female=2
Age	<60 years=1,≥60 years =2
Body mass index(BMI, kg/m^2^)	<18.5=1, 18.5-23.9=2, 24-27.9=3, ≥28=4
Marry status	Married=1,Single/Divorced/Widowed=2
Education level	Elementary school and below=1,Abover elementary school=2
Monthly household income (yuan)	≤3000=1,>3000 yuan =2
Residential situation	With family=1,Others=2
Smoking status	Smokers/quit smokers =1,Never=2
Drinking status	Drinkers/quit drinkers=1,Never=2
Employment	Yes=1,No=2
Falls	Yes=1,No=2
Dialysis duration	<5 years=1,≥5years=2
Exercise	No=1,Yes=2
Dialysis frequency	Twice a week=1,Three times a week=2,Five times two weeks=3
Vascular access	Arteriovenous fistula=1,Artificial blood vessels=2,Central venous catheter=3
Hypertension	Yes=1,No=2
Diabetes	Yes=1,No=2
Heart disease	Yes=1,No=2
Cerebral disease	Yes=1,No=2
Other diseases	Yes=1,No=2
Self-efficacy(fen)	Low level=1, Middle level=2, High level=3
Frailty grades	Robust=0,Pre-frailty=1,Frailty=2

## Ethical considerations

This study was approved by the biomedical ethics committee of Xiangyang No.1 People’s Hospital (NO. XYYYE20240025). Written informed consent was waived by the ethics committee since this study involves only the completion of a questionnaire; verbal informed consent from the research subjects is required before they can complete the questionnaire. However, no signature is required from the research subjects. After obtaining informed consent, distribute the questionnaire via “Questionnaire Star” (an online survey platform) on the spot for participants to fill in the relevant content, and the time recorded by Questionnaire Star shall be regarded as the time of consent to fill in. This study adhered to the principles outlined in the Declaration of Helsinki. Clinical trial number: not applicable.

## Statistical analysis approach

The study was statistically analyzed using IBM SPSS 25.0 and Amos 26.0. Descriptive statistics (means and frequency percentages) and analytical statistics (Pearson correlation and model construction) were employed. Independent samples t-tests or one-way analysis of variance (ANOVA) were utilized to compare the different frailty grades based on demographic features. Pearson correlation analysis was conducted to explore the relationship between psychological resilience, frailty, and self-efficacy. To test the hypothesis that psychological resilience mediated the relationship between frailty and self-efficacy, the bootstrap method was used for mediation effect analysis and model construction by Amos 26.0, with the significance of the mediation model assessed using the bias-corrected percentile bootstrap method (replicate sampling of 2000, 95% CI). Model fitting was evaluated using the following indices: CMIN/DF (Chi-Square to Degrees of Freedom Ratio), CFI (comparative fit index), IFI (incremental fit index), NFI (normed fit index), TLI (Tucker–Lewis index), GFI (goodness-of-fit index), AGFI (adjusted goodness-of-fit index), RMSEA (root mean square error of approximation), and SRMR (standardized root mean square residual). The level of statistical significance was set at less than 0.05 (two-tailed).

## Results

### Demographic and clinical characteristics of the study population by categories of different frailty grades

The demographic and clinical characteristics of the patients are presented in **Table 2**. In this study, a total of 408 participants were recruited for the study, resulting in a final participation count of 397. 53.1% were male and 46.9% female. Patients aged 60 years or older accounted for 48.6%. Overweight patients constituted 30.5%. A significant majority of participants with a history of falls was 84.4%, and most participants had not been employed (88.7%). Approximately 83.9% of the patients were married, and 90.2% lived with family. More than half of the participants had an elementary school diploma or a lower level of education (54.7%). The economic status of the participants was predominantly average low (52.9%). Over 72.0% of the participants hardly exercised, while only 28.0% exercised regularly. Regarding dialysis duration, 65.2% of participants had been undergoing dialysis for 5 years or less, and 34.8% for 5 years or more. Hypertension was the most prevalent disease among the participants, occurring more than twice as frequently as other diseases. The majority of participants with arteriovenous fistulas were 78.8%.

**Table 2:** Differences of demographic and clinical characteristics between different frailty grades in HD participants(n= 397).

Variables	Total (n = 397)	Robust (n = 141)	Pre-frailty (n = 104)	Frailty (n = 152)	*p*	statistic
Sex, n (%)	nan	nan	nan	nan	0.480	1.468
Male	211 (53.1)	75 (53.2)	60 (57.7)	76 (50)		
Female	186 (46.9)	66 (46.8)	44 (42.3)	76 (50)		
Age(years), n (%)	nan	nan	nan	nan	0.002	12.681
<60	204 (51.4)	84 (59.6)	59 (56.7)	61 (40.1)		
≥60	193 (48.6)	57 (40.4)	45 (43.3)	91 (59.9)		
BMI(kg/m^2^), n (%)	nan	nan	nan	nan	0.183	8.834
<18.5	56 (14.1)	24 (17)	13 (12.5)	19 (12.5)		
18.5-23.9	220 (55.4)	84 (59.6)	56 (53.8)	80 (52.6)		
24-27.9	88 (22.2)	24 (17)	22 (21.2)	42 (27.6)		
≥28	33 ( 8.3)	9 (6.4)	13 (12.5)	11 (7.2)		
Marry status, n (%)	nan	nan	nan	nan	0.153	3.752
Married	333 (83.9)	121 (85.8)	81 (77.9)	131 (86.2)		
Single/Divorced/Widowed	64 (16.1)	20 (14.2)	23 (22.1)	21 (13.8)		
Falls, n (%)	nan	nan	nan	nan	< 0.001	27.019
Yes	335 (84.4)	135 (95.7)	88 (84.6)	112 (73.7)		
No	62 (15.6)	6 (4.3)	16 (15.4)	40 (26.3)		
Employment, n (%)	nan	nan	nan	nan	0.598	1.027
Yes	45 (11.3)	19 (13.5)	11 (10.6)	15 (9.9)		
No	352 (88.7)	122 (86.5)	93 (89.4)	137 (90.1)		
Education level, n (%)	nan	nan	nan	nan	0.113	4.356
Elementary school and below	217 (54.7)	70 (49.6)	54 (51.9)	93 (61.2)		
Abover elementary school	180 (45.3)	71 (50.4)	50 (48.1)	59 (38.8)		
Monthly household income(yuan), n (%)	nan	nan	nan	nan	0.071	5.293
≤3000	210 (52.9)	64 (45.4)	57 (54.8)	89 (58.6)		
>3000	187 (47.1)	77 (54.6)	47 (45.2)	63 (41.4)		
Residentialsituation, n (%)	nan	nan	nan	nan	0.791	0.468
With family	358 (90.2)	128 (90.8)	92 (88.5)	138 (90.8)		
Others	39 ( 9.8)	13 (9.2)	12 (11.5)	14 (9.2)		
Smoking status, n (%)	nan	nan	nan	nan	< 0.001	13.87
Smokers/quit smokers	115 (29.0)	26 (18.4)	31 (29.8)	58 (38.2)		
Never	282 (71.0)	115 (81.6)	73 (70.2)	94 (61.8)		
Drinking status, n (%)	nan	nan	nan	nan	0.090	4.806
Drinkkers/quit drinkers	144 (36.3)	42 (29.8)	38 (36.5)	64 (42.1)		
Never	253 (63.7)	99 (70.2)	66 (63.5)	88 (57.9)		
Exercise, n (%)	nan	nan	nan	nan	< 0.001	18.421
Yes	111 (28.0)	52 (36.9)	35 (33.7)	24 (15.8)		
No	286 (72.0)	89 (63.1)	69 (66.3)	128 (84.2)		
Heartdisease, n (%)	nan	nan	nan	nan	< 0.001	17.743
Yes	108 (27.2)	22 (15.6)	29 (27.9)	57 (37.5)		
No	289 (72.8)	119 (84.4)	75 (72.1)	95 (62.5)		
Diabetes, n (%)	nan	nan	nan	nan	< 0.001	16.38
Yes	134 (33.8)	33 (23.4)	32 (30.8)	69 (45.4)		
No	263 (66.2)	108 (76.6)	72 (69.2)	83 (54.6)		
Hypertension, n (%)	nan	nan	nan	nan	0.200	3.217
Yes	330 (83.1)	122 (86.5)	88 (84.6)	120 (78.9)		
No	67 (16.9)	19 (13.5)	16 (15.4)	32 (21.1)		
Dialysis duration(years), n (%)	nan	nan	nan	nan	0.094	4.731
<5	259 (65.2)	94 (66.7)	75 (72.1)	90 (59.2)		
≥5	138 (34.8)	47 (33.3)	29 (27.9)	62 (40.8)		
Dialysis frequency, n (%)	nan	nan	nan	nan	0.713	2.124
Twice a week	48 (12.1)	18 (12.8)	11 (10.6)	19 (12.5)		
three times a week	224 (56.4)	77 (54.6)	56 (53.8)	91 (59.9)		
five times two weeks	125 (31.5)	46 (32.6)	37 (35.6)	42 (27.6)		
Vascular access, n (%)	nan	nan	nan	nan	0.002	16.855
Arteriovenous fistula	313 (78.8)	121 (85.8)	86 (82.7)	106 (69.7)		
Artificial blood vessels	38 ( 9.6)	11 (7.8)	4 (3.8)	23 (15.1)		
Central venous catheter	46 (11.6)	9 (6.4)	14 (13.5)	23 (15.1)		
Self-efficacy(fen), Median (IQR)	6.5 (5.0, 8.2)	7.2 (5.3, 8.3)	6.5 (5.2, 8.0)	5.8 (4.2, 7.8)	0.002	12.196
Self-efficacy(fen), n (%)	nan	nan	nan	nan	0.004	15.312
<5	98 (24.7)	27 (19.1)	20 (19.2)	51 (33.6)		
5-6.9	123 (31.0)	38 (27)	37 (35.6)	48 (31.6)		
≥7	176 (44.3)	76 (53.9)	47 (45.2)	53 (34.9)		
Symptom management(fen), Median (IQR)	6.5 (4.8, 8.2)	7.2 (5.5, 8.5)	6.5 (5.0, 8.2)	5.8 (4.0, 8.0)	0.002	12.068
Common disease management(fen), Median (IQR)	6.5 (5.0, 8.5)	7.0 (5.5, 8.5)	6.5 (5.4, 8.1)	6.0 (4.5, 8.0)	0.008	9.688
Psychological resilience(fen), Median (IQR)	23.0 (20.0, 30.0)	26.0 (20.0, 30.0)	22.0 (19.8, 30.0)	22.5 (19.0, 30.0)	0.001	13.121

Patients with frailty were older, smoked more, and had a shorter duration of exercise, diabetes mellitus (DM), heart disease, and cerebral disease than those in the pre-frailty and robust groups (*p* < 0.05; **Table 2**).

The total score of patient self-efficacy and its dimensions, including symptom management and common disease management, were shown in **Table 2**. The total score of patient psychological resilience was 23.0 (20.0, 30.0). Patients with frailty had lower self-efficacy and psychological resilience than those in the pre-frailty and robust groups.

### Correlations among psychological resilience, frailty and self-efficacy

**Table 3** reveals correlations among psychological resilience, frailty, and self-efficacy in patients with HD. A negative correlation was observed between frailty and self-efficacy (r = -0.166, *p* < 0.01). Additionally, frailty was negatively correlated with psychological resilience (r = -0.222, *p* < 0.01). Conversely, the results indicated a positive correlation between self-efficacy and psychological resilience (r = 0.287, *p* < 0.01). Although the effect sizes for these results were small, they demonstrated statistically significant differences. These findings suggest that as patients’ frailty increases, their tendency towards negative psychological resilience may decrease, which may in turn lower their self-efficacy.

**Table 3:** Correlation among frailty, self efficacy, and psychological resilience.

	Frailty	Self efficacy	Psychological resilience	Symptom management	Common disease management
Frailty	1				
Self-efficacy	-0.166**	1			
Psychological resilience	-0.222**	0.287**	1		
Symptom management	-0.167**	0.986**	0.289**	1	
Common disease management	-0.151**	0.942**	0.259**	0.873**	1

*p< 0.05; **p≤ 0.01.

### Testing the hypothesis model

**Figure 2:**
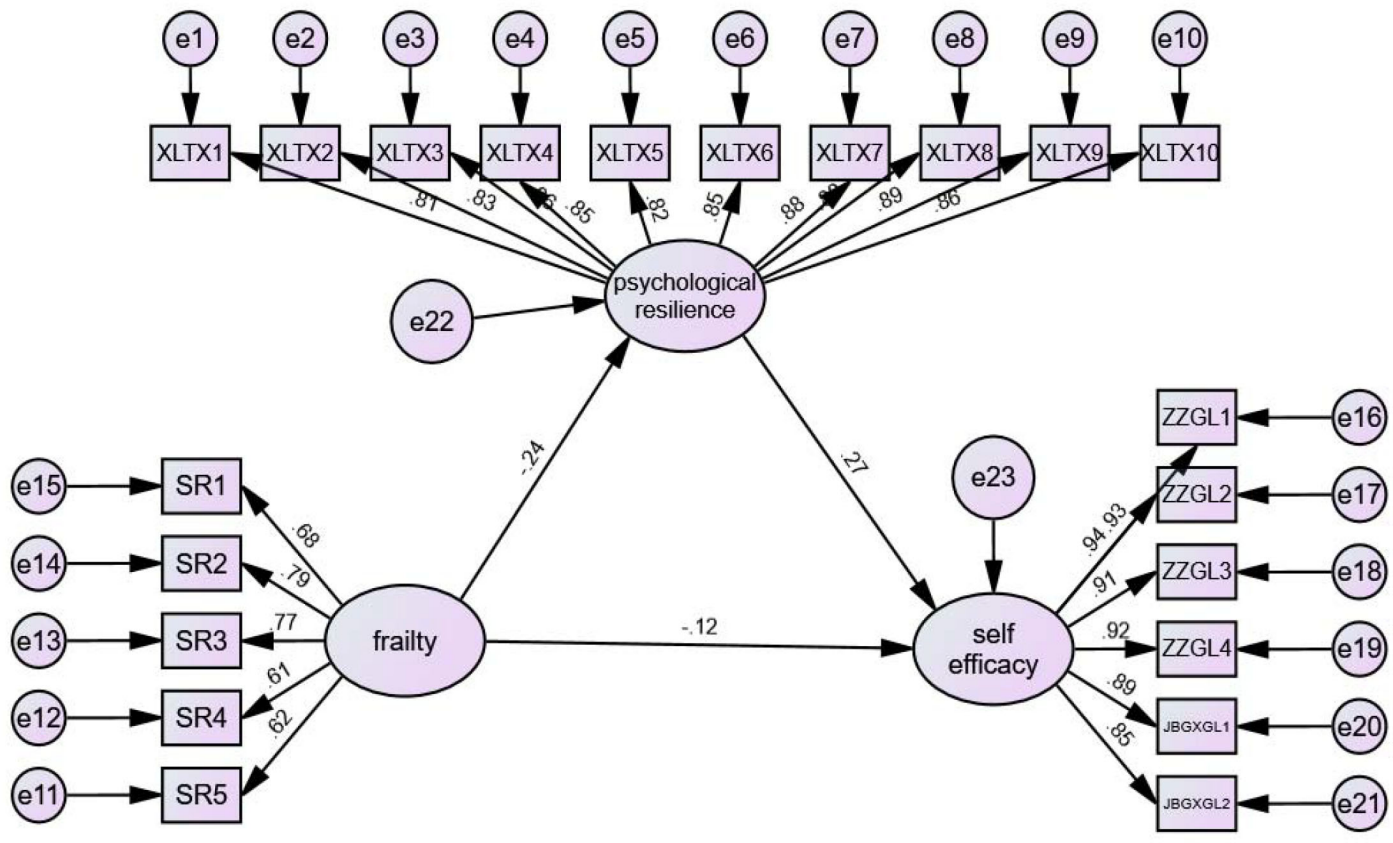
The SEM of predictors of psychological resilience, self efficacy and frailty.

From the analysis of the three correlations, a correlation was identified among psychological resilience, frailty, and self-efficacy, thus enabling the performance of mediation effect analysis. Following the validation procedure for mediating effects, this study selected AMOS 26.0 software to construct a model. In the SEM analysis aimed at determining the direct and indirect relationships between frailty and self-efficacy, with psychological resilience serving as a mediating variable, all paths were found to be significant. In the self-efficacy scale, the modification indices for item 6⟷ item 5 were 14.690 and 20.134, respectively-both exceeding the critical value of 11.34 (*p* < 0.001). These two items both assess individuals’ confidence in “managing chronic disease without relying on medication or medical visits, through personal behavioral control.” Their shared variance is not fully captured by the latent construct “chronic disease self-efficacy” (e.g., aspects such as “proactive self−management” or “consistent cognition of non-pharmacological interventions”). This aligns with the theoretical rationale for residual correlation modification, rendering the correlated residuals theoretically justifiable. After incorporating these modifications, the overall fit indices of the structural equation model improved (**Table 4**). Overall quality of fit statistics indicated that the proposed model was a good fit, with a Chi-square Degrees of Freedom Ratio (CMIN/DF) of 2.837, a Root Mean Square Error of Approximation (RMSEA) of 0.068, a Goodness of Fit Index (GFI) of 0.881, an Adjusted Goodness of Fit Index (AGFI) of 0.851, and a Comparative Fit Index (CFI) of 0.957. **Figure 2** illustrates that the direct relationship between frailty and self-efficacy was significant (*β* = 0.124, *p* = 0.026). Furthermore, the indirect relationship between the two variables, mediated by psychological resilience, was also significant (*β* = -0.244, *β=*0.267, *p ≤* 0.001), suggesting that psychological resilience acts as a partial mediation effect between patients’ frailty and self-efficacy.

**Table 4:** Model fitting index of structural equation model.

Fit Index	The Goodness-of-Fit Index of SEM								
	CMIN/DF	CFI	IFI	NFI	TLI	RMSEA	SRMR	GFI	AGFI
Ideal standards	<5.00	>0.90	>0.90	>0.90	>0.90	<0.08	<0.08	>0.90	>0.90
Measurement value	3.286	0.946	0.946	0.924	0.939	0.076	0.05	0.862	0.829
Revised Measurement value	2.837	0.957	0.957	0.935	0.951	0.068	0.064	0.881	0.851

CMIN/DF, Chi-square fit statistics/degree of freedom; CFI, comparative fit index. IFI, incremental fit index; NFI, normed fit index; TLI, Tucker–Lewis index; RMSEA, root mean square error of approximation; SRMR, standardized root mean square residual; GFI, goodness-of-fit index; AGFI, adjusted goodness-of-fit index.

### Path testing for structural equation model

In the path analysis of structural equation modeling, as depicted in **Table 5**, a significant correlation is observed between frailty and psychological resilience (*p* < 0.001), as well as between resilience and self-efficacy (*p* < 0.001). Hypothesis 1 and Hypothesis 2 were valid. The table reveals that frailty accounts for 24.4% of the variance in psychological resilience, albeit with an inverse relationship. In contrast, psychological resilience explains 26.7% of the variance in self-efficacy. However, in the association between frailty and self-efficacy, despite being statistically significant (*p* = 0.026), only 12.4% of the variance is accounted for, again with an inverse relationship. Hypothesis 3 was valid.

**Table 5:** Standard and non-standard coefficients in the proposed path mode.

Path relationship test	Std	Unstd	S.E.	C.R.	*P*
Frailty→Psychological resilience	-0.244	-0.587	0.139	-4.237	***
Psychological resilience→Self-efficacy	0.267	0.887	0.174	5.091	***
Frailty→Self-efficacy	-0.124	-0.986	0.445	-2.219	0.026

***p≤ 0.001.Std., standardized; Unstd., unstandardized; S.E., standard error; C.R.,Critical Ratio.

### Mediating effects of psychological resilience

Indirect relationships among psychological resilience, frailty, and self-efficacy were examined using a bootstrap method with 2000 resamples and a bias-corrected Percentile confidence interval of 95%. **Table 6** reveals that patient frailty has a direct impact on self-efficacy, with a direct effect size of -0.986 (95% CI, -1.958 to -0.107), while psychological resilience serves as the part of mediating variable between frailty and self-efficacy, with an indirect effect size of -0.521 (95% CI, -0.899 to -0.236). The effect percentage calculations in **Table 6** indicate that psychological resilience, as a mediating factor, accounts for an indirect effect size of 35%, whereas the direct effect size of patient frailty constitutes 65%. Hypothesis 4 was valid. Consequently, psychological resilience exerts an indirect influence on the association between frailty and self-efficacy.

**Table 6:** Bootstrap mediated effects results.

Parameter	Estimate	Lower	Upper	*P*	Efficiency ratio (%)
Indirect effects	-0.521	-0.899	-0.236	0.001	35%
Direct effects	-0.986	-1.958	-0.107	0.026	65%
Total effects	-1.507	-2.533	-0.612	0.001	100%

## Discussion

Consistent with the study’s hypothesis, the results indicated a negative correlation between frailty and all domains of the perceived self-efficacy questionnaire, including symptom management and common disease management, as well as psychological resilience. Conversely, there was a positive correlation between all domains of the perceived self-efficacy questionnaire and psychological resilience. Furthermore, psychological resilience served as a partial mediation effect between frailty and self-efficacy. Notably, the associations reported in the SEM are unadjusted for covariates and have methodological limitations; in contrast, the supplementary multivariate regression analyzes provide more rigorous evidence for the associations among frailty, psychological resilience, and self-efficacy by controlling for confounding variables (**supplementary material**). We noted that the GFI and AGFI values below 0.90 are primarily due to the two indices’ sensitivity to sample size and model complexity, as well as the slightly lower measurement precision of frailty compared to psychological resilience and self-efficacy (**supplementary material**). This may be attributed to the fact that the frailty scale used is self-reported with only 5 items, which impacts construct validity. Future studies will incorporate objective indicators to enhance measurement precision. However, core fit indices (e.g., CFI, RMSEA) all meet the recommended criteria, indicating satisfactory overall model fit without compromising its validity. In this study, we used COR theory to explain how frailty affects self-efficacy. Our results show that frailty may diminish psychological resilience, thereby reducing self-efficacy. This study provides an empirical basis for understanding the frailty - self-efficacy link and new insights into the cascading effects of frailty’s complexity.

Frailty, characterized by diminished physiological reserves and resilience to stress associated with aging (38, 39), significantly increases the risk of negative health outcomes among patients with ESRD patients. Frailty fundamentally arises as the outcome of prolonged depletion of resources and directly impairs patients’ physical, functional, and psychological resource reserves. The primary factors influencing frailty encompass gender, age, obesity, low income, marital status, physical dysfunction, peripheral vascular disease, heart disease, diabetes, albumin levels, sarcopenia, cognitive impairment, and depression (39–45). Self-efficacy constitutes a core constituent of personal resources. The higher the incidence of frailty, the more resources are depleted; over time, this leads to the impairment of self-efficacy (36). Our research discovered that frailty is inversely related to all domains of the perceived self-efficacy questionnaire, including symptom management and common disease management, in patients undergoing HD. Consistent with earlier studies, general self-efficacy negatively predicts frailty in older individuals (36, 46).

The results indicated a negative correlation between frailty and psychological resilience in HD. Zhang B et al. (47) found that low psychological resilience was a primary factor associated with frailty in renal transplant recipients. Similarly, Sini M Stenroth et al. (48) reported that higher resilience was linked to lower frailty levels in older adults. Resilience, defined as the ability to adapt appropriately in adversity, trauma, tragedy, threats, or significant stress, is a personal trait or adaptive process that can be developed (49). In other words, psychological resilience helps individuals proactively mobilize, reorganize, and preserve existing resources (e.g., emotional regulation and problem-solving strategies) when their resources are threatened, thereby mitigating the negative effects of resource depletion. The loss of physical resources associated with frailty is perceived as a “resource threat,” which can lead to emotional stress, anxiety, and a decline in self-worth. Therefore, individuals with higher psychological resilience can appraise this threat and mitigate the loss of external resources by mobilizing internal resources (such as optimism and problem−solving abilities) (50) and by seeking external support (family, healthcare teams, peers) (51) to replenish the depleted resources. Higher resilience levels are associated with a decreased likelihood of chronic pain, improved daily and physical function, better quality of life, enhanced psychosocial functioning, and a reduced risk of co-morbid mental health disorders (52). Therefore, patients with greater psychological resilience tend to cope better with physical, psychological, and social challenges.

Our study revealed that psychological resilience may have a positive correlation with the self-efficacy of HD patients. Self-efficacy is a protective factor of resilience (53). Xu Y et al. (54) found that resilience positively impacted the self-efficacy and creativity of participants. Conversely, self-efficacy influences the role of psychological resilience in some studies. Parviniannasab AM et al. (53) suggested that promoting diabetes management self-efficacy could effectively enhance resilience and reduce diabetes distress. Increased resilience is associated with qualities such as spirituality, humor, hope, and spiritual influences, which are essential components of resilience in stress reduction, with self-efficacy serving as a key element (55). Individuals with higher self-efficacy exhibit more resilience attributes, making them better equipped to handle stress (56). Dialysis patients face significant physiological, psychological, and socio-economic pressures. Psychological resilience helps patients make full use of their resources to regulate emotions and face various stresses positively, encourages them to proactively learn dialysis-related knowledge and adhere to self-management behaviors (such as diet and exercise) (57), and enables more effective utilization of support from family and healthcare professionals (17, 58), thereby enhancing their sense of self−efficacy and their quality of life.

Psychological resilience, as a mediating variable, influences multiple independent and dependent variables. Wang Y et al. (59) showed that among pregnant women in China, self-efficacy’s impact on prenatal stress is mediated by social support and resilience. Tian Y et al. (60)explained resilience’s mediating role in the relationship between fear of progression and sleep quality in hematological malignancy patients. Resilience has been proven to improve sleep quality. Upasen R et al. (61)demonstrated that resilience mediates the relationship between social support and mental health. The mediating analysis confirmed resilience’s role as a mediator between perceived stress and symptoms of anxiety, depression, and psychological distress (62). Another study showed that resilience partially mediated the relationship between frailty and depression in older adults living with HIV (63). Resilience leads to greater positive emotions, which in turn reduce daily pain catastrophizing (64). In summary, psychological resilience plays an important role in the treatment of diseases. In this study, psychological resilience functions as a resource regulator, providing both “resource buffering” and “resource rebuilding.” It transforms or compensates for resource loss. Frailty depletes resources and generates stress; through positive regulation of emotions, behaviors, and social support, it enhances patients’ sense of efficacy regarding self-management behaviors, thereby serving to protect and restore resources.

Older adults and family caregivers reported that physical health declines with age and perseverance levels determine frailty. Understanding resilience in this context will help nurses facilitate the use of individual and sociocultural resources to improve resilience experiences among older adults. Various coping strategies, such as maintaining active involvement in health management and social life, can enhance self-efficacy and build resilience in older adults (65). Based on COR theory, psychological resilience acts as a “resource bridge” between frailty and self-efficacy. By weakening this bridge (reducing psychological resilience), debility may ultimately deplete self-efficacy, a core psychological resource. However, frailty is not only common among the elderly but is also increasingly prevalent in younger patients. Therefore, future research should explore the role of resilience training in reducing frailty incidence and enhancing self-efficacy in HD patients. Thus, clinical trial studies are recommended to further evaluate the collateral effects of psychological resilience and frailty on patients’ self-efficacy.

## Limitations

This study was a cross-sectional investigation and could not establish causal relationships, presenting several limitations. Firstly, data collected from a single region. The participants, comprising 397 patients undergoing HD, were conveniently recruited from three hospitals in Xiangyang. This sampling strategy may limit the generalizability of the results. To address this, future research should consider using randomized sampling and a time-series design. Besides, cultural disparities across different countries and regions may restrict the generalizability of the current research findings. Secondly, this study did not fully account for the impact of confounding variables on the model, potentially introducing social desirability bias. Additionally, it is important to note that a significant limitation of this study was the deterioration of patients during dialysis, complications from blood pressure drops, and the impatience of elderly patients, which made it challenging to complete the questionnaire. Consequently, we administered the questionnaire within 2 hours of initiating dialysis treatment. Furthermore, this study was conducted exclusively on patients undergoing HD, and it is suggested that similar studies be extended to patients receiving other forms of renal replacement therapy, such as peritoneal dialysis or kidney transplantation. Comparing HD, peritoneal dialysis, and kidney transplantation could offer a deeper understanding of these concepts. Finally, since the use of self-reported scales alone is insufficient for comprehensively understanding a patient’s condition, gaining deeper insights into their self-management skills, and exploring the psychosocial issues faced by patients receiving HD, it is recommended to conduct further research through open-ended interviews or related qualitative studies.

## Conclusion

Guided by the COR theory, the path relationship among frailty, psychological resilience, and self-efficacy follows the sequence: frailty (resource threat), psychological resilience (resource regulation), and self-efficacy (sense of behavioral mastery), with a negative correlation between the former two and a positive correlation between the latter two. Psychological resilience, as a mediating variable, plays a certain role in chronic disease management and the promotion of patients’ health behaviors. Furthermore, psychological resilience may have been shown to bolster self-efficacy in HD patients. Self-efficacy, in turn, may enhance patients’ cognitive and emotional performance, decrease mortality and hospitalization rates, and ultimately improve treatment adherence and modify health behaviors. Consequently, given the regulatory impact of psychological resilience, practical steps can be implemented to mitigate frailty, enhance self-efficacy, and better manage the disease in HD patients.

## Data Availability

The original contributions presented in the study are included in the article/**Supplementary Material**. Further inquiries can be directed to the corresponding authors.
