# Supplementary material for: The mediating role of psychological resilience in the relationship between frailty and self-efficacy among dialysis patients
Source: Front Psychiatry. 2026 Jan 12;16:1542031. doi: 10.3389/fpsyt.2025.1542031 (PMC12833567; doi:10.3389/fpsyt.2025.1542031)
Supplement: Supplementary file 1 [file DataSheet1.docx]

**Supplementary material.**

| **Table1. Construct reliability and convergent validity test.** | | | | | |
| --- | --- | --- | --- | --- | --- |
| **Latent variables** | **Observed variables** | **Factor loadings** | **Cronbach’s alpha** | **AVE** | **CR** |
| Frailty | SR1 | 0.684 | 0.824 | 0.488 | 0.825 |
|  | SR2 | 0.792 |  |  |  |
|  | SR3 | 0.767 |  |  |  |
|  | SR4 | 0.607 |  |  |  |
|  | SR5 | 0.622 |  |  |  |
| Psychological resilience | XLTX1 | 0.806 | 0.964 | 0.727 | 0.964 |
|  | XLTX2 | 0.831 |  |  |  |
|  | XLTX3 | 0.861 |  |  |  |
|  | XLTX4 | 0.848 |  |  |  |
|  | XLTX5 | 0.824 |  |  |  |
|  | XLTX6 | 0.851 |  |  |  |
|  | XLTX7 | 0.876 |  |  |  |
|  | XLTX8 | 0.875 |  |  |  |
|  | XLTX9 | 0.892 |  |  |  |
|  | XLTX10 | 0.856 |  |  |  |
| Self-efficacy | SE1 | 0.930 | 0.966 | 0.827 | 0.966 |
|  | SE2 | 0.943 |  |  |  |
|  | SE3 | 0.913 |  |  |  |
|  | SE4 | 0.923 |  |  |  |
|  | SE5 | 0.891 |  |  |  |
|  | SE6 | 0.853 |  |  |  |

SR1-5 represent the five items of the Simple Frail Scale. XLTX1-10 represent the ten items of the Connor-Davidson Resilience Scale. SE1-6 represent the six items of the self-efficacy scale.

| **Table2. Discriminant Validity Test.** | | | |
| --- | --- | --- | --- |
|  | **Psychological resilience** | **Self-efficacy** | **Frailty** |
| Psychological resilience | 0.488 |  |  |
| Self-efficacy | 0.297 | 0.727 |  |
| Frailty | -0.244 | -0.189 | 0.827 |
| Square Root of AVE | 0.853 | 0.909 | 0.699 |

| **Table3. Multivariate linear regression of frailty predicting self‑efficacy.** | | | | |
| --- | --- | --- | --- | --- |
| **Variable** | **crude.Coefficient_95CI** | **crude.P_value** | **adj.Coefficient_95CI** | **adj.P_value** |
| (Intercept) | 6.82 (6.45~7.19) | <0.001 | 6.89 (6.14~7.64) | <0.001 |
| Frailty |  |  |  |  |
| Robust | ref |  | ref |  |
| Pre-frailty | -0.36 (-0.94~0.21) | 0.216 | -0.32 (-0.88~0.23) | 0.258 |
| Frailty | -0.91 (-1.43~-0.4) | 0.001 | -0.62 (-1.16~-0.09) | 0.023 |
| Sex, n (%) |  |  |  |  |
| Male | ref |  | ref |  |
| Female | -0.38 (-0.83~0.07) | 0.096 | -0.42 (-0.85~0.02) | 0.061 |
| Age(years), n (%) |  |  |  |  |
| <60 | ref |  | ref |  |
| ≥60 | 0.18 (-0.27~0.64) | 0.423 | 0.22 (-0.23~0.68) | 0.340 |
| Marry status, n (%) |  |  |  |  |
| Married | ref |  | ref |  |
| Single/Divorced/Widowed | -0.97 (-1.58~-0.37) | 0.002 | -0.93 (-1.53~-0.33) | 0.003 |
| Monthly household income(yuan), n (%) |  |  |  |  |
| ≤3000 | ref |  | ref |  |
| >3000 | 0.51 (0.06~0.96) | 0.027 | 0.33 (-0.12~0.78) | 0.147 |
| Exercise, n (%) |  |  |  |  |
| No | ref |  | ref |  |
| Yes | 0.79 (0.29~1.29) | 0.002 | 0.51 (0.02~1.01) | 0.043 |
| Vascular access, n (%) |  |  |  |  |
| Arteriovenous fistula | ref |  | ref |  |
| Artificial blood vessels | -1.62 (-2.37~-0.86) | <0.001 | -1.32 (-2.06~-0.57) | 0.001 |
| Central venous catheter | 0.19 (-0.51~0.88) | 0.595 | 0.35 (-0.35~1.04) | 0.329 |
| Hypertension, n (%) |  |  |  |  |
| No | ref |  | ref |  |
| Yes | 0.21 (-0.39~0.82) | 0.488 | 0.01 (-0.56~0.58) | 0.972 |
| Diabetes, n (%) |  |  |  |  |
| No | ref |  | ref |  |
| Yes | -1 (-1.47~-0.54) | <0.001 | -0.74 (-1.22~-0.27) | 0.002 |
| Heart disease, n (%) |  |  |  |  |
| No | ref |  | ref |  |
| Yes | 0.11 (-0.4~0.61) | 0.685 | 0.27 (-0.23~0.76) | 0.292 |

Variables with a univariate *P*-value < 0.05 have been adjusted: sex+age+hypertension+heart disease+marry status+monthly house hold income+exercise+vascular access+diabetes.

| **Table4. Multivariate linear regression of psychological resilience predicting self‑efficacy.** | | | | |
| --- | --- | --- | --- | --- |
| **Variable** | **crude.Coefficient_95CI** | **crude.P_value** | **adj.Coefficient_95CI** | **adj.P_value** |
| (Intercept) | 4.32 (3.61~5.03) | <0.001 | 4.89 (3.89~5.89) | <0.001 |
| psychologicalresilience | 0.08 (0.06~0.11) | <0.001 | 0.07 (0.04~0.1) | <0.001 |
| Sex, n (%) |  |  |  |  |
| Male | ref |  | ref |  |
| Female | -0.38 (-0.83~0.07) | 0.096 | -0.32 (-0.74~0.11) | 0.148 |
| Age(years), n (%) |  |  |  |  |
| <60 | ref |  | ref |  |
| ≥60 | 0.18 (-0.27~0.64) | 0.423 | 0.24 (-0.21~0.68) | 0.297 |
| Marry status, n (%) |  |  |  |  |
| Married | ref |  | ref |  |
| Single/Divorced/Widowed | -0.97 (-1.58~-0.37) | 0.002 | -0.66 (-1.26~-0.07) | 0.030 |
| Monthly household income(yuan), n (%) |  |  |  |  |
| ≤3000 | ref |  | ref |  |
| >3000 | 0.51 (0.06~0.96) | 0.027 | 0.33 (-0.11~0.77) | 0.139 |
| Exercise, n (%) |  |  |  |  |
| No | ref |  | ref |  |
| Yes | 0.79 (0.29~1.29) | 0.002 | 0.36 (-0.13~0.85) | 0.147 |
| Vascular access, n (%) |  |  |  |  |
| Arteriovenous fistula | ref |  | ref |  |
| Artificial blood vessels | -1.62 (-2.37~-0.86) | <0.001 | -1.35 (-2.08~-0.63) | <0.001 |
| Central venous catheter | 0.19 (-0.51~0.88) | 0.595 | 0.3 (-0.38~0.97) | 0.389 |
| Hypertension, n (%) |  |  |  |  |
| No | ref |  | ref |  |
| Yes | 0.21 (-0.39~0.82) | 0.488 | 0 (-0.55~0.56) | 0.988 |
| Diabetes, n (%) |  |  |  |  |
| No | ref |  | ref |  |
| Yes | -1 (-1.47~-0.54) | <0.001 | -0.72 (-1.19~-0.26) | 0.002 |
| Heart disease, n (%) |  |  |  |  |
| No | ref |  | ref |  |
| Yes | 0.11 (-0.4~0.61) | 0.685 | 0.32 (-0.16~0.81) | 0.194 |

Variables with a univariate *P*-value < 0.05 have been adjusted: sex+age+hypertension+heart disease+marry status+monthly house hold income+exercise+vascular access+diabetes.

| **Table5. Multivariate linear regression analysis of frailty and psychological resilience.** | | | | |
| --- | --- | --- | --- | --- |
| Variable | crude.Coefficient_95CI | crude.P_value | adj.Coefficient_95CI | adj.P_value |
| (Intercept) | 26.08 (25~27.17) | <0.001 | 27.91 (24.44~31.38) | <0.001 |
| frailty | -0.93 (-1.33~-0.53) | <0.001 | -0.59 (-1.02~-0.16) | 0.008 |
| Sex, n (%) |  |  |  |  |
| Male | ref |  | ref |  |
| Female | -1.67 (-3.2~-0.14) | 0.033 | -0.32 (-2.03~1.4) | 0.718 |
| Age(years), n (%) |  |  |  |  |
| <60 | ref |  | ref |  |
| ≥60 | -1.5 (-3.03~0.03) | 0.055 | -0.28 (-1.88~1.32) | 0.729 |
| Marry status, n (%) |  |  |  |  |
| Married | ref |  | ref |  |
| Single/Divorced/Widowed | -3.89 (-5.94~-1.84) | <0.001 | -3.66 (-5.69~-1.63) | <0.001 |
| Education level |  |  |  |  |
| Elementary school and below | ref |  | ref |  |
| Abover elementary school | 2.15 (0.63~3.68) | 0.006 | 1.07 (-0.48~2.63) | 0.176 |
| Smoking status |  |  |  |  |
| Smokers/quit smokers | ref |  | ref |  |
| Never | -2.08 (-3.67~-0.5) | 0.01 | -1.81 (-3.61~-0.02) | 0.049 |
| Monthly household income(yuan), n (%) |  |  |  |  |
| ≤3000 | ref |  | ref |  |
| >3000 | 1.87 (0.34~3.4) | 0.017 | 0.33 (-1.22~1.89) | 0.675 |
| Exercise, n (%) |  |  |  |  |
| No | ref |  | ref |  |
| Yes | 4.33 (2.67~5.98) | <0.001 | 2.61 (0.93~4.28) | 0.002 |
| Employment |  |  |  |  |
| Yes | ref |  | ref |  |
| No | -3.89 (-6.28~-1.5) | 0.002 | -1.09 (-3.61~1.42) | 0.395 |
| Falls |  |  |  |  |
| Yes | ref |  | ref |  |
| No | -2.53 (-4.63~-0.43) | 0.019 | -0.95 (-3.02~1.12) | 0.37 |
| Hypertension, n (%) |  |  |  |  |
| No | ref |  | ref |  |
| Yes | 1.27 (-0.78~3.31) | 0.225 | 0.38 (-1.54~2.3) | 0.697 |
| Diabetes, n (%) |  |  |  |  |
| No | ref |  | ref |  |
| Yes | -2.3 (-3.91~-0.69) | 0.005 | -1.46 (-3.04~0.11) | 0.069 |
| Heart disease, n (%) |  |  |  |  |
| No | ref |  | ref |  |
| Yes | -3.21 (-4.9~-1.51) | <0.001 | -2.01 (-3.66~-0.36) | 0.017 |

Variables with a univariate *P*-value < 0.05 have been adjusted: sex+age+marry status+education level+drinking status+monthly house hold income+exercise+employment+falls+hypertension+diabetes+heart disease.
